# *IRS1*, *TCF7L2*, *ADRB1*, *PPARG*, and *HHEX* Polymorphisms Associated with Atherogenic Risk in Mexican Population

**DOI:** 10.1155/2013/394523

**Published:** 2013-11-25

**Authors:** B. I. Estrada-Velasco, M. Cruz, V. Madrid-Marina, G. A. Martínez-Nava, J. Gomez-Zamudio, A. I. Burguete-García

**Affiliations:** ^1^Centro de Enfermedades Infecciosas, Instituto Nacional de Salud Pública, Avenida Universidad 655, Santa María Ahuacatitlán, Cuernavaca, 62100 Cuernavaca, MOR, Mexico; ^2^Unidad de Investigación Médica en Bioquímica, Hospital de Especialidades, IMSS, Avenida Cuauhtémoc 330, Doctores, Cuauhtémoc, CMN Siglo XXI, 06720 México, DF, Mexico

## Abstract

*Objective*. We aimed to explore the association between polymorphisms of *IRS1* (rs1801278), *TCF7L2* (rs7903146 and rs12255372), *ADRB1* (rs1801253), *PPARG* (rs1801282), and *HHEX* (rs5015480) genes with atherogenic risk (AI = Total cholesterol/HDL) in MetS, T2D, and healthy populations from the Mexican Social Security Institute. *Methodology and Results*. Four hundred thirty-five MetS, 517 T2D, and 547 healthy individuals were selected. The association between the SNPs and the atherogenic index was evaluated by multiple linear regression and multinomial logistic regression models. The *ADRB1* gene showed a statistically significant association with high-risk atherogenic index, OR = 2.94 (IC 95% 1.64–5.24; *P* < 0.0001) for the Arg/Gly variant, under the dominant model an OR = 2.96 (IC 95% 1.67–5.25; *P* < 0.0001), and under the Log additive model an OR = 2.52 (IC 95% 1.54–4.15; *P* < 0.0001). *Conclusions*. The Arg389Gly polymorphism of the *ADRB1* gene may be a worthy biological marker to predict the risk of developing cardiovascular diseases given a high-risk atherogenic index.

## 1. Introduction

The metabolic syndrome (MetS) is a group of metabolic disorders that directly promote the development of cardiovascular diseases (CVD) [1, 2]. One of these disorders, insulin resistance (IR) [3], is at the core of the physiopathology of the MetS and type 2 diabetes (T2D). Lipid metabolism is affected by the consequences of IR, culminating in the MetS characteristic lipid disorders: IR leads to high levels of very low density lipoproteins (VLDL) that cause high triglyceride levels and low HDL-c levels, a profile known as atherogenic lipoprotein phenotype [4]. IR can, thus, be associated with fatty acid metabolism disequilibrium [5].

The last two decades have seen a marked increase in the number of people with MetS worldwide [6]. In Mexico, the prevalence of MetS under the ATP III, AHA/NHLBI, and IDF criteria was 36.8%, 41.6%, and 49.8%, respectively [7].

Several susceptibility loci for MetS component traits have been soundly identified to play a role in the risk of IR [8], obesity [9, 10], hypertriglyceridemia, and low HDL-c levels [11, 12]. The IRS1 gene Gly972Arg polymorphism affects the PI3-k/AKT/GSK3 signaling pathway, producing IR due to a decrease in the ability of insulin to transport glucose, translocation of the glucose transporter, and glycogen synthesis [13]. This polymorphism has been associated with elevated serum triglyceride, low HDL-c levels, high free fatty acid levels, and elevated systolic blood pressure [14]. Variants in the TCF7L2, SLC30A8, *HHEX*, FTO, PPARG, and KCNJ11 genes are associated with T2D and cardiovascular disease risk [15–17]. Polymorphisms in the beta-adrenergic receptors genes are associated with cardiovascular and metabolic phenotypes. The *β* adrenergic receptors play a major role in energy expenditure through thermogenesis stimulation [18]. The aim of this study was to analyze the association between the IRS1 (rs1801278), TCF7L2 (rs7903146 and rs12255372), ADRB1 (rs1801253), PPARG (rs1801282), and *HHEX* (rs5015480) polymorphisms with IR, lipid profile, and atherogenic risk.

## 2. Methodology

### 2.1. Design and Study Population

In a cross-sectional study, we recruited 431 patients with MetS and 517 patients with T2D defined according to the criteria of the American Heart Association/National Heart, Lung, and Blood Institute Scientific Statement (AHA/NHLBI) [2] and the American Diabetes Association (ADA), respectively, and 547 health individuals (without MetS or T2D) between 35 and 65 years old. The AHA/NHLBI criteria are: waist circumference ≥102 cm for men and ≥88 cm for women, fasting glucose ≥100 mg/dL, systolic blood pressure (SBP) ≥130 mm Hg, diastolic blood pressure (DBP) ≥85 mm Hg, HDL-c <40 mg/dL for men and <50 mg/dL for women, and triglycerides ≥150 mg/dL. The study population was selected at the IMSS Medical Center of Mexico City. The inclusion criterion for healthy individuals was absence of a family history of T2D among parents, siblings, or offspring. Written consent was obtained from the participants, and the protocol was approved by the Institutional Review Board (IRB) of the National Public Health Institute and the National Committee of the IMSS.

### 2.2. Biochemical and Anthropometric Measurements

All participants answered a questionnaire used for collecting socioeconomic data, as well as family history of diabetes and other diseases. The following anthropometric measurements were performed: weight (kg), height (cm), body mass index (BMI) (kg/m^2^), waist (cm) and hip (cm) circumference, and the waist/hip ratio (WHR). SBP and DBP were measured with a mercury column sphygmomanometer (American Diagnostic Inc., New York), after the patients had rested and were seated for at least 5 minutes. A blood sample was obtained from all participants, after a 12-hour fast, for the determination of biochemical profiles of glucose (mg/dL), total cholesterol, LDL cholesterol (LDL-C, mg/dL), HDL cholesterol (HDL-C, mg/dL), and triglycerides (mg/dL). These parameters were determined by the ILab 30 Clinical Chemistry System instrument (Instrumentation Laboratory, Barcelona, Spain). IR was estimated by means of the Homeostasis Model Assessment-Insulin Resistance, mmol/pmol (HOMA-IR), and the atherogenic index (AI) was estimated with the following formula: AI = Total cholesterol/HDL-c, with a cut-off point for cardiovascular risk of >4.5 for women and >5 for men [19].

### 2.3. Genotyping

DNA extraction from peripheral vein blood samples was carried out by a semiautomated method with the commercial QIAamp DNA Blood Mini/Kit column purification kits (Qiagen, Germany). DNA integrity was determined by 0.8% agarose gel electrophoresis, stained with ethidium bromide, and visualized through the Gel Doc 2000 equipment (BIORADCA, USA). DNA concentration was determined by spectrophotometry, using the VICTOR^3^ 1420 equipment (Perkin-Elmer, Germany). Single nucleotide polymorphism (SNP) analysis was carried out in duplicate with real-time PCR TaqMan technology (Applied Biosystems 7900HT equipment, Foster City, California, USA), following standardized techniques. Studied SNPs were: *IRS1* (rs1801278), *TCF7L2* (rs7903146 and rs12255372), *ADRB1 *(rs1801253), *PPARG *(rs1801282), and *HHEX* (rs5015480) (Table 1). For quality control (QC) of the genotyping, we used a call rate of 0.99 for cases and controls; all the samples with 0.99 call rate were analyzed by two independent researchers who scored genotype results using the graphical view (SD 2.1.1, Applied Biosystems), and all differences were resolved by discussions between the independent researchers and by rerunning the samples if necessary. If the concordance was less than 99.99%, this was considered as a missing value. Only 3.35% of the samples had less than 99.99% concordance.

In order to control the possible effect of population stratification, a panel of ancestry informative markers (AIM) was also genotyped. The panel was integrated by 27 AIMS (rs2814778, rs723822, rs1008984, rs1435090, rs17203, rs768324, rs719776, rs1112828, rs1403454, rs3340, rs2077681, rs1935946, rs1320892, rs1373302, rs2695, rs1980888, rs1327805, rs2207782, rs1487214, rs2078588, rs724729, rs292932, rs1369290, rs386569, rs718092, rs878825, and rs16383) taken from a previous assessed ancestry panel [20]. These markers showed differences in allele frequency between European 30%, African 5%, and Amerindian populations 65%, in Mexican population in a previous report of our research group [20].

### 2.4. Statistical Analysis

In order to compare the relevant variants between cases and controls, the Kruskal-Wallis test for group comparison was used for continuous variables, and the chi square test (*χ* ^2^) was used for categorical data. Hardy-Weinberg equilibrium was evaluated for each variant under study. A multiple linear regression analysis was performed to evaluate the effects of the selected variants on the IR biochemical markers (glucose, insulin, and HOMA-IR) and the atherogenic profile biomarkers (total cholesterol, LDL-c, HDL-c, and triglycerides). A multinomial logistic regression was also carried out to evaluate the effect of each and every variant on AI risk (AI = Total cholesterol/HDL; with the following cut-off points: low risk <4.5 for women and <5 for men, medium risk 4.5–7 for women and 5–9 for men, and high risk >7 for women and >9 for men) [18] for the different genotypes in the codominant, dominant, recessive, and log-additive heritability models. All models were adjusted for age, sex, disease status, BMI, and ancestry percentage. The study's power average was 80%. The possibility of multiple testing burden was avoided by using the Bonferroni method (0.05/42 comparisons = *P* ≤ 0.001 for linear regression and 0.05/12 comparisons = *P* ≤ 0.004 for multinomial logistic regression) and false-positive report probability (FPRP) for correction of multiple comparisons [21]. The STATA SE version 12.0 statistical package software (College Station, TX) was used for the analyses.

## 3. Results

The general characteristics of the groups studied are shown in Table 2. The Low risk AI group had lower levels in mg/dL to total cholesterol (190.93 ± 39.92, *P* = 0.0001), LDL (119.21 ± 30.88, *P* = 0.0001), triglycerides (158.95 ± 77.54, *P* = 0.0001), glucose (118.41 ± 56.90, *P* = 0.07), and insulin (11.73 ± 8.89, *P* = 0.009), compared to medium and high risk AI groups. There was no difference in BMI between the three groups. The ancestry percentage was similar in the three groups, with the Amerindian group having the highest percentage, followed by the European group, whereas the African group had a minimum percentage. 

Table 3 shows that all studied polymorphisms are in Hardy-Weinberg equilibrium. For all polymorphisms, genotype and allele frequencies were similar in the three groups of risk AI. All polymorphisms were in HW equilibrium in the control group of low risk AI (*IRS1 P* = 0.95, *TCF7L2* rs12255372 *P* = 0.42, rs7903146 *P* = 0.16, *PPARG P* = 0.14, *ADRB1 P* = 0.31, and *HHEX* *P* = 0.95). 

In order to evaluate the variant's effect on the IR biochemical and on atherogenic profile biomarkers, measured by total cholesterol, LDL-c, HDL-c, triglycerides, glucose, insulin, and HOMA-IR, a multiple linear regression analysis was carried out according to the codominant, dominant, and recessive models, adjusted for age, sex, BMI, ancestry percentage, and disease status. No statistically significant associations were found (data not shown).

A possible association between the polymorphic variants and other characteristics such as SBP, DBP, and WHR, was assessed through a multiple linear regression analysis adjusted for age, sex, BMI, ancestry percentage, and disease status. However, the results of this analysis did not show any potential association (data not shown).

Table 4 shows multinomial logistic regression results that evaluates the effect of each and every variant on the risk of presenting an AI category (medium or high risk; low risk was considered the reference category) for the log-additive model, adjusted for age, sex, BMI, disease status, and ancestry percentage; only the *ADRB1* polymorphism (Arg389Gly) showed a statistically significant association with high-risk AI (OR= 2.52, IC 95% 1.54–4.15; *P* < 0.0001). 

Finally, the multinomial logistic regression results that evaluate the effect *ADRB1* variant on the risk of presenting an AI category (medium or high risk; low risk was considered the reference category), for the codominant, dominant, and recessive models, adjusted for age, sex, BMI, disease status, and ancestry percentage, showed a statistically significant association of the *ADRB1* gene with high-risk AI, (OR = 2.94, IC 95% 1.64–5.24; *P* < 0.0001) for the Arg/Gly variant, for the dominant model an OR = 2.96 (IC 95% 1.67–5.25; *P* < 0.0001) (Table 5).

## 4. Discussion and Conclusions

Identification of the genes involved in the metabolic alterations produced in complex diseases like MetS should be a priority to study. We include in the study SNPs of genes related to glucose metabolism [22, 23], fatty acid metabolism, and development of an atherogenic profile in MetS patients.

The main findings of this study are that the *ADRB1* gene heterozygote and dominant model showed an association with high risk AI, with an OR of 2.94 (IC 95% 1.64–5.24; *P* < 0.0001) for the Arg/Gly variant, for the dominant model an OR of 2.96 (IC 95% 1.67–5.25; *P* < 0.0001), and for the Log additive model an OR of 2.52 (IC 95% 1.54–4.15; *P* < 0.0001). 

The *ADRB1* gene association is in agreement with reports that associate this gene variant with fat accumulation, BMI, and obesity [18]. The significant association of Arg389Gly variant means that carriers of these polymorphisms are almost three times more likely to present a high-risk AI than noncarriers. These associations are independent of age, sex, BMI, and ancestry percentage. These observations are supported by studies that associated the variant with a poor coupling of the receptor with the G-protein, which translates to functional consequences [24]. Associations between the *ADRB1* gene Arg389Gly polymorphism and heart diseases and an increase in fat accumulation have also been found [25].

The *HHEX* T/C heterozygote and the dominant model showed an association although not significant with an increase in total cholesterol levels and in LDL-c levels, suggesting that carriers of this variant may be prone to develop lipid metabolism alterations. This is consistent with recent results for the Mexican population [23]. The *HHEX* gene regulates pancreatic development by controlling endodermic cell proliferation and the positioning of the cells that will later turn into pancreatic tissue. This probably occurs through the effects of *HHEX* on the transcription and translation of the cell proliferator. *HHEX* expression remains activated in the adult pancreas, which suggests that *HHEX* may have additional functions in differentiated pancreatic cells. Because the *HHEX* gene plays a role in pancreas development, as described above, it may be involved in glucose metabolism [25]. Some *HHEX* gene variants have been strongly associated with T2D and insulin-stimulated glucose release [26].

The results of the evaluation of the effects of the polymorphic variants on the IR biochemical markers (glucose, insulin, and HOMA-IR) showed that the *IRS1 *Gly/Arg variant is associated although not significant with an increase in HOMA-IR, consistentl with previous reports of an association between the *IRS1* gene Gly972Arg polymorphism and IR, and with a higher risk of T2D and CVD in various populations, including the Mexican group, where this association was replicated [27–29]. Some studies suggest that this polymorphism may contribute to IR development due to impairment in the insulin ability to activate the IRS1/PI3-kinase/Akt/GSK-3 signaling pathway, causing deficient glucose transportation, translocation of glucose transporters, and glycogen synthesis [28].

We found no significant association with PPAR*γ* Pro12Ala polymorphism; this is contrary to previous studies which report that Ala12 homozygotes have an increased risk of cardiovascular disease (CAD), but a meta-analysis conducted to investigate the relationship between the PPAR*γ* polymorphism and the potential risk of CAD reported that this risk is higher in Caucasian populations compared to a Asiatic population, suggesting that the risk for cardiovascular disease conferred by the Pro12Ala polymorphism differs across ethnic populations [30].

Even TCF7L2 may contribute not only to the development of T2DM but also to the development of CAD because its important role in vascular remodeling through the regulation of smooth muscle cell proliferation and endothelial cell growth [31, 32]; in our study, we did not find significant association. Our result is similar to a recent report by the Atherosclerosis Risk in Communities (ARIC) study where they did not find significant associations between TCF7L2 SNPs and incident vascular events [33].

The results of this assessment of the effects of the polymorphic variants on the medium or high risk of atherogenesis confirm the association between the *ADRB1* gene Arg389Gly [17]. Our results suggest that the *ADRB1* gene Arg389Gly polymorphism can be a good biological marker to predict the risk of cardiovascular diseases development given a high-risk AI. This is in agreement with the notion that the genetic susceptibility to atherosclerosis is due in part to known metabolic risk factors (in this case dyslipidemia) [34]. Further studies of this genetic effect are required to evaluate its potential interaction with other risk factors involved in the same metabolic pathway, since the known genetic risk factors do not fully explain the hereditary propensity to atherosclerosis [34].

## Figures and Tables

**Table 1:** SNP, rs number, and GenBank number.

SNP	rs	GenBank no.
IRS1	rs1801278	S62539
TCF7L2	rs7903146	NT_030059 ABBA01063000
TCF7L2	rs12255372	NT_030059 ABBA01063000
ADRB1	rs1801253	NT_030059.12 ABBA01062983
PPARG	rs1801282	U79012 015869 ABBA01025204
HHEX	rs5015480	NT_030059.12 ABBA01021892

**Table 2:** Sociodemographics, clinics, and ancestry characteristics.

Variables	Low risk AI (711)	Medium risk AI (728)	High risk AI (56)	*P* value
Age	48.17 ± 8.61	46.86 ± 7.98	44.68 ± 7.98	0.0009*
Total cholesterol (mg/dL)	190.93 ± 39.92	222.63 ± 51.35	261.84 ± 65.86	0.0001*
LDL-c (mg/dL)	119.21 ± 30.88	140.08 ± 37.32	156.14 ± 50.88	0.0001*
HDL-c (mg/dL)	49.93. ± 12.97	38.89 ± 10.44	29.59 ± 7.36	0.0001*
Triglycerides (mg/dL)	158.95 ± 77.54	263.56 ± 160.32	361.75 ± 246.11	0.0001*
Disease status (%)				
Health	45.71	28.16	30.36	<0.0001**
Metabolic syndrome	17.02	39.56	39.29	
Type 2 diabetes	37.37	32.28	30.36	
BMI	28.75 ± 4.47	29.15 ± 4.14	29.48 ± 5.43	0.07*
SBP (mm Hg)	119.28 ± 13.42	120.65 ± 12.33	119.21 ± 10.79	0.03*
DBP (mm Hg)	75.5 ± 8.63	76.10 ± 8.15	77.46 ± 7.99	0.21*
Glucose (mg/dL)	118.41 ± 56.90	127.29 ± 72.19	120.14 ± 56.96	0.07*
Insulin (pmol/L)	11.73 ± 8.89	12.27 ± 7.28	13.44 ± 9.23	0.009*
HOMA-IR	3.73 ± 5.23	4.07 ± 4.96	4.20 ± 4.32	0.005*
Ancestry contribution				
African	0.03 ± 0.021	0.03 ± 0.023	0.03 ± 0.030	0.69*
European	0.32 ± 0.11	0.33 ± 0.10	0.32 ± 0.09	0.58*
Amerindian	0.65 ± 0.12	0.64 ± 0.11	0.65 ± 0.10	0.55*
Sex (%)				
F	47.12	40.66	57.14	0.007**
M	52.88	59.34	42.86	
Educational level (%)				
Elementary or lower	31.16	30.70	31.91	0.88**
Middle school	27.08	28.07	31.91	
High school	19.21	20.61	21.28	
College or higher	22.54	20.61	14.89	

Data shown in means and SD. F: female, M: male, SBP: systolic blood pressure, DBP: diastolic blood pressure. *Kruskal Wallis *P* value, **Chi square *P* value.

**Table 3:** Genotype frequencies and Hardy-Weinberg equilibrium.

	Medium risk AI		High risk AI		Controls	
	*n*	%	*n*	%	*n*	%
IRS1 (rs1801278) Gly972Arg						
G/G	6570	92.46	477	90.4	6377	92.70
G/A	51	7.17	5	9.6	49	7.13
A/A	3	0.42	0	0	1	0.14
G		0.96		0.96		0.95
A		0.04		0.04		0.05

HW* (*P* value)						**0.95**

TCF7L2 (rs12255372)						
G/G	507	74.99	343	70.8	512	75.85
G/T	156	23	131	27.1	149	22.10
T/T	14	2.1	1	2.1	14	2.07
G		0.86		0.85		0.87
T		0.13		0.15		0.12

HW (*P* value)						**0.42**

TCF7L2 (rs7903146)						
C/C	477	72.96	33	66	466	72.83
C/T	179	23.47	12	24	180	24.58
T/T	24	3.57	5	10	22	2.59
C		0.83		0.78		0.83
T		0.17		0.25		0.17

HW (*P* value)						**0.17**

PPARG (rs1801282) Pro12Ala						
C/C	556	78.2	37	78.7	502	73.8
C/G	142	20	8	17	159	23.4
G/G	13	1.8	2	4.2	19	2.8
C		0.88		0.87		0.86
G		0.12		0.13		0.14

HW (*P* value)						**0.14**

ADRB1 (rs1801253) Arg389Gly						
C/C	555	76.6	32	57.1	557	78.8
C/G	157	21.75	23	41.1	144	20.4
G/G	12	1.6	1	1.8	6	0.8
C		0.88		0.78		0.89
G		0.12		0.22		0.11

HW (*P* value)						**0.31**

HHEX (rs5015480)						
T/T	267	37.8	18	32.7	266	37.36
T/C	341	48.3	28	50.9	316	47.93
C/C	98	13.9	9	16.4	93	14.71
T		0.62		0.58		0.61
C		0.38		0.42		0.38

HW (*P* value)						**0.95**

*HW: Hardy-Weinberg equilibrium.

**Table 4:** Association of polymorphisms with atherogenic index.

SNP	Medium risk AI	*P *	High risk AI	*P *
	ORa (CI)		ORa (CI)	
IRS1 rs1801279				
Log additive	1.00 (0.68, 1.47)	0.99	1.24 (0.49, 3.13)	0.65

TCF7L2 rs12255372				
Log additive	0.95 (0.76, 1.19)	0.67	1.15 (0.64, 2.08)	0.63

TCF7L2 rs7903146				
Log additive	0.93 (0.76, 1.15)	0.51	1.35 (0.82, 2.21)	0.24

PPARG rs1801282				
Log additive	0.81 (0.65, 1.01)	0.06	0.89 (0.48, 1.65)	0.71

**ADRB1 rs1801253**				
Log additive	1.16 (0.91, 1.47)	0.22	**2.52** **(1.54, 4.15) **	<0.0001*

HHEX rs5015480				
Log additive	1.03 (0.88, 1.22)	0.68	1.24 (0.82, 1.87)	0.30

Adjusted for age, sex, BMI, disease status, and ancestry percentage. IA Low risk was the reference category. *Bonferroni corrected *P* value threshold *P* ≤ 0.004.

Bold text denotes significant *P* values (<0.05).

**Table 5:** Association of polymorphism ADRB1 rs1801253 with atherogenic index.

SNP	Medium risk AI	*P *	High risk AI	*P *
	ORa (CI)		ORa (CI)	
**C/G**	1.08 (0.83, 1.41)	0.55	**2.94 (1.64, 5.24)**	<0.0001*
G/G	2.19 (0.81, 5.95)	0.12	3.40 (0.39, 29.61)	0.27
^ d^ **C/G + G/G**	1.12 (0.87, 1.46)	0.36	**2.96 (1.67, 5.25)**	<0.0001*
^ r^G/G	2.16 (0.79, 5.84)	0.13	2.47 (0.29, 21.27)	0.40
Log additive	1.16 (0.91, 1.47)	0.22	**2.52 (1.54, 4.15) **	<0.0001*

Adjusted for age, sex, BMI, Disease status, and ancestry percentage. IA Low risk was the Reference category. *Bonferroni corrected *P* value threshold *P* ≤ 0.025. ^d^Dominant model. ^r^Recessive model.

Bold text denotes significant *P* values (<0.05).

## Abbreviations

AI: Atherogenic index
AIM: Ancestry informative markers
BMI: Body mass index
CVD: Cardiovascular diseases
DBP: Diastolic blood pressure
HDL: High density lipoproteins
HOMA-IR: Homeostasis model assessment-insulin resistance
IR: Insulin resistance
LDL: Low density lipoproteins
MetS: Metabolic syndrome
SBP: Systolic blood pressure
T2D: Type 2 diabetes
VLDL: Very low density lipoproteins.
